# Learning spatiotemporal statistical shape models for non-linear dynamic anatomies

**DOI:** 10.3389/fbioe.2023.1086234

**Published:** 2023-01-27

**Authors:** Jadie Adams, Nawazish Khan, Alan Morris, Shireen Elhabian

**Affiliations:** ^1^ School of Computing, University of Utah, Salt Lake City, UT, United States; ^2^ Scientific Computing and Imaging Institute, University of Utah, Salt Lake City, UT, United States

**Keywords:** statistical shape modeling, spatiotemporal modeling, cardiac motion, nonlinear dynamics, population morphology analysis

## Abstract

Numerous clinical investigations require understanding changes in anatomical shape over time, such as in dynamic organ cycle characterization or longitudinal analyses (e.g., for disease progression). Spatiotemporal statistical shape modeling (SSM) allows for quantifying and evaluating dynamic shape variation with respect to a cohort or population of interest. Existing data-driven SSM approaches leverage information theory to capture population-level shape variations by learning correspondence-based (landmark) representations of shapes directly from data using entropy-based optimization schemes. These approaches assume sample independence and thus are unsuitable for sequential dynamic shape observations. Previous methods for adapting entropy-based SSM optimization schemes for the spatiotemporal case either utilize a cross-sectional design (ignoring within-subject correlation) or impose other limiting assumptions, such as the linearity of shape dynamics. Here, we present a principled approach to spatiotemporal SSM that relaxes these assumptions to correctly capture population-level shape variation over time. We propose to incorporate modeling the underlying time dependency into correspondence optimization *via* a regularized principal component polynomial regression. This approach is flexible enough to capture non-linear temporal dynamics while encoding population-specific spatial regularity. We demonstrate our method’s efficacy on synthetic data and left atrium segmented from cardiac MRI scans. Our approach better captures the population modes of variation and a statistically significant time dependency than existing methods.

## 1 Introduction

Statistical shape models (SSMs) provide a compact representation of shape in relation to a population. SSM is a valuable tool in clinical research because it allows for quantifying and analyzing anatomical shape variation with respect to a cohort of interest. SSM has been effectively used to quantify group differences (for example, between healthy and disease-specific populations) and in downstream tasks such as pathology detection and disease diagnosis (Bhalodia et al. (2020); Harris et al. (2013); Atkins et al. (2017); Gaffney et al. (2019)). Many clinical investigations require *spatiotemporal* evaluation, i.e., analysis of anatomical shape change over time. Such is the case for studies of *dynamic* motion that involve dense observations over short time intervals (such as organ cycles), as well as for *longitudinal* studies that involve sparse observations over extended periods (such as in disease staging and intervention analysis). Traditional SSM methods are incapable of representing spatiotemporal data and can only be applied in a time-agnostic manner by assuming a *cross-sectional* study design. Here individual subject correlation across time is ignored, and each time point is incorrectly assumed to be an independent observation, i.e., a different subject. Disregarding the inherent correlation of shapes from the same sequence can confound the resulting population statistics and subsequent analyses (Gerig et al. (2016); Fitzmaurice and Ravichandran (2008)). Spatiotemporal SSM captures the time-based trajectory of shapes across patient sequences (intra-subject variation) and the population (inter-subject variation).

**FIGURE 1:**
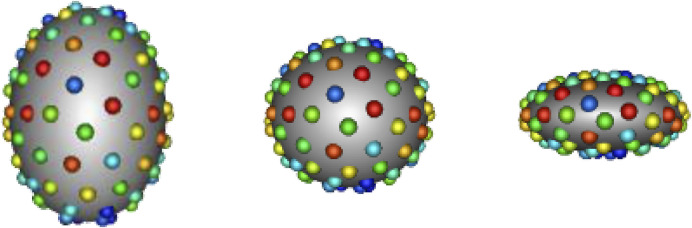
Example PDM: An example of a PDM with 128 particles on three ellipsoid shapes, where color denotes correspondence. The particle color pattern matches across shapes, indicating geometrically consistent particle placement.

In SSM, a shape can be represented explicitly or implicitly. The explicit representation comprises of sets of landmark or correspondence points, i.e., geometrically consistent points defined on each anatomical surface in the shape population. The implicit representation takes the form of deformation fields or coordinate transformations in relation to a predefined shape atlas (Miller et al. (2014); Cootes et al. (2004)). Explicit correspondence-based SSM is one of the most popular techniques due to the simplicity and interpretability of the shape representation (Cerrolaza et al. (2019)). Correspondence points can be easily interpreted and visualized by clinicians. Additionally, this approach does not require the formulation of an atlas, which is non-trivial to define. For these reasons, we focus on correspondence-based SSM in this work. Historically, correspondence points were defined manually by domain experts to capture biologically relevant features (Bookstein (1996); Dryden and Mardia (2016)). However, such manual annotation was burdensome, subjective, and sparse—potentially missing clinically relevant shape attributes. More recently, computational methods have been utilized automatically define dense sets of correspondence points, or *point distribution models* (PDMs), to represent shape. A small example of a PDM can be seen in Figure 1.

Automatic PDM construction is computationally derived by formulating point placement as an optimization problem. Parametric optimization schemes have been formulated which utilize a geometric basis, i.e., spherical harmonics (assuming a template sphere) (Styner et al. (2006)), wavelet-based (Nain et al. (2007)), and functional maps (Ovsjanikov et al. (2012)). Optimization schemes that do not rely on parameterization but rather utilize a point-based representation have also been formulated using metrics such as entropy (Cates et al. (2007)) and minimum description length (Davies et al. (2002)). These approaches avoid complex parameterization construction steps and the limitations inherent in parametric representations, such as restriction to specific topologies and bias resulting from the choice of basis or template. The non-parametric techniques have been shown to produce more robust and compact models that better retain clinically relevant shape characteristics (Goparaju et al. (2018); Goparaju et al. (2022)). In this work, we utilize the entropy-based approach to PDM optimization formulated in Cates et al. (2007), a. k.a. particle-based shape modeling, as it is a data-driven approach that does not require any form of atlas. Correspondence point, or *particle*, positions are optimized directly from shape data to capture population-level shape variations. This formulation is implemented in the open source software, ShapeWorks (Cates et al. (2017)), and explained in detail in Section 2.2.1. ShapeWorks SSM has been proven to be state-of-the-art (Goparaju et al. (2018)) and has been successfully used in a variety of medical applications, including downstream tasks such as pathology detection and disease diagnosis (Bhalodia et al. (2020); Harris et al. (2013); Atkins et al. (2017)).

ShapeWorks cannot be directly applied to spatiotemporal data without assuming a cross-sectional design, where instances from a temporal sequence are treated independently. Adams et al. (2022) proposed adapting the entropy-based PDM optimization objective to disentangle subject and time dependencies. This disentangled technique (explained further in Section 2.2.2) outperforms an image-based approach that was originally proposed in Morris et al. (2020) for estimating organ segmentation and functional measurements over time. Adams et al. (2022) adapted this image-based approach for spatiotemporal SSM by first generating a PDM for a single, corresponding time point across subjects, then independently propagating the correspondence points across individual time sequences using image-based deformable registration. The disentangled entropy formulation provided an improvement over this image-based method, both in terms of capturing shape variation and the underlying time dependency. However, this approach still assumes a Gaussian distribution to approximate subject-wise entropy across time, hence violating the independence assumption. Furthermore, it does not explicitly parameterize the time dependency and requires consistent, identical time points for every subject in the cohort, limiting its utility in practical medical settings.

Datar et al. (2009) proposed incorporating regression analysis in the process of optimizing correspondences. The details of this approach are explained in Section 2.2.3. Linear regression allowed for directly modeling the time-dependency and including partial sequences; however, it is limited to cases of linear shape dynamics. This technique may be applicable to specific studies of developmental modeling that involve linear growth, but it is not applicable to the general, much more common case of non-linear shape dynamics or longitudinal changes. The motion of the left atrium, for example, is an instance of highly non-linear dynamics as the volume increases and decreases cyclically. Datar et al. (2012) proposed using linear mixed effect modeling rather than simple regression in spatiotemporal PDM optimization. This hierarchical model allowed for capturing the global population trend as a fixed effect and individual trends as random effects. However, it is still limited to the case of linear longitudinal changes. Both the linear regression and mixed effects methods further suffered from the limitation of not handling spatial correlations between points on a shape (i.e., spatial regularity). In these methods, each particle coordinate is regressed independently without providing smoothness constraints.

In this paper, we introduce a novel approach to spatiotemporal SSM that combines entropy-based PDM optimization with non-linear regression to model the time dependency. Specifically, we extend the method presented by Cates et al. (2007) to incorporate regularized polynomial regression analysis in particle optimization. This regression is fit in the principal component subspace that best explains the data span to leverage population-specific statistics for capturing the spatial regularity of shape dynamics across time. The benefits of our proposed approach to spatiotemporal PDM optimization can be outlined as follows.

• It provides both inter-subject shape correspondence across the population and temporal intra-subject correspondence across time points without incorrect independence assumptions.
• It directly models the time-dependency in a manner that is not only flexible and non-linear, but also regularized to be generalizable and to reflect population-specific statistics.
• It does not require temporal sequences to be consistent across subjects. Thus subjects with partial observations or missing time points can be included in PDMs generated *via* the proposed approach.

The proposed method is capable of modeling any case of dynamic or longitudinal shape, surpassing the limitations of existing, aforementioned spatiotemporal SSM methods and increasing the potential for SSM to provide medical insight. The code will be released to magnify the scientific impact and clinical utility of the proposed method. Section 2, provides the details of the method as well as an explanation of three baseline methods used for comparison: the cross-sectional PDM formulation presented in Cates et al. (2007), the disentangled intra- and inter-subject entropy approach presented in Adams et al. (2022), and the linear regression approach presented in Datar et al. (2009). In Section 3, we first utilize a synthetic dataset to provide evidence of the theoretical motivation for the proposed method, then demonstrate its efficacy on a real dataset. We utilize a population of left atrium sequences over the cardiac cycle from CINE magnetic resonance imaging (MRI) scans to demonstrate the benefits of our approach over the comparison methods in capturing non-linear shape dynamics. The left atrium is an example of dynamic motion; however, our method also applies to longitudinal studies. We employ quantitative and qualitative metrics to verify the superiority of the proposed method.

## 2 Methods

### 2.1 Notation

**FIGURE 2:**
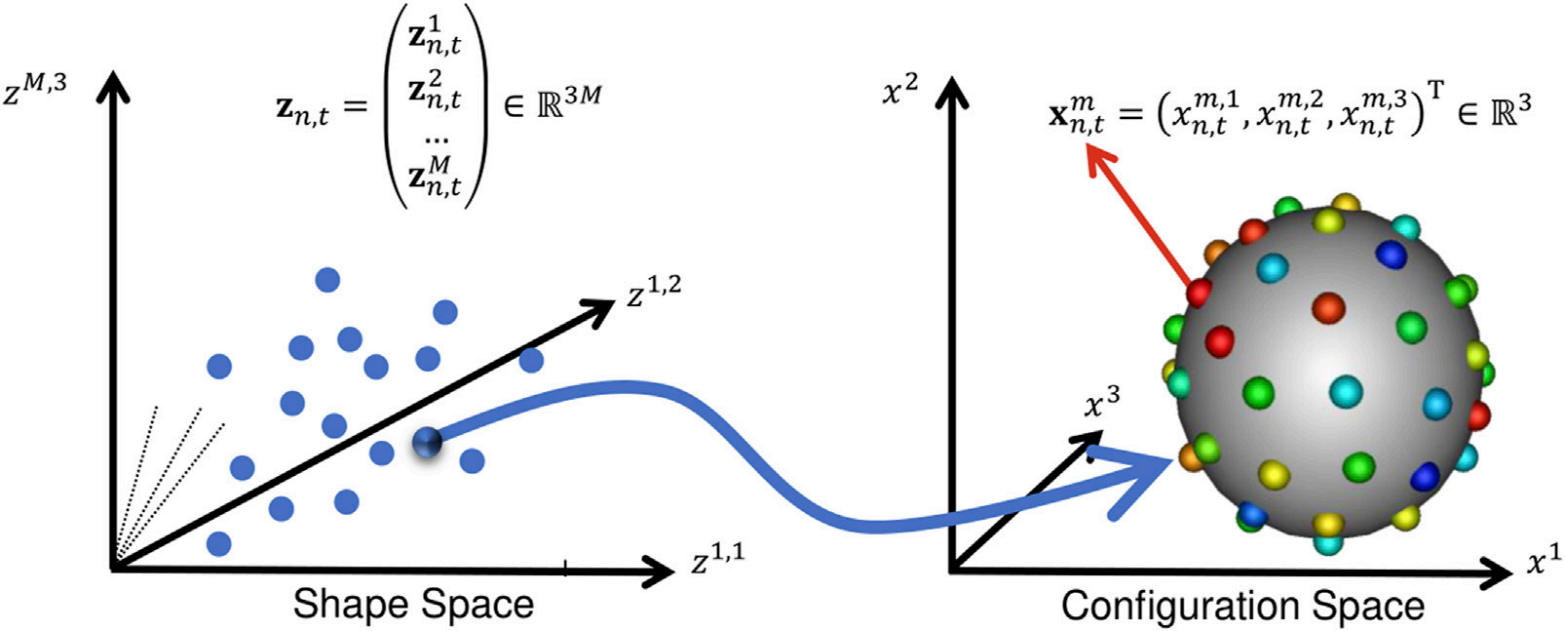
Configuration Space vs. Shape Space: This illustration aids in understanding the notation in Section 2. A point in shape space corresponds to a particle set in configuration space.

For spatiotemporal SSM, we define a dataset of *N* subjects each with a consistent time-sequence of *T* − shapes, each represented as a set of *d* − dimensional correspondence points (or particles). In this work, shape is segmented from volumetric images, so *d* = 3. To optimize particle position, we define two forms of random variables: configuration and shape space variables. These two spaces are illustrated in Figure 2.

The configuration space variable captures sample-specific geometry. It is denoted **X**_*n*,*t*_, representing the particle position on the *n* − th subject at the *t* − th time point, where *n* ∈ [1, *N*] and *t* ∈ [1, *T*]. *M* − realizations of this random variable defines the point set (or PDM) of the *n*, *t* − shape: $x_{n,t}=\left[x_{n,t}^{1},x_{n,t}^{2},\ldots ,x_{n,t}^{M}\right]\in R^{dM}$ , where a single particle $x_{n,t}^{m}\in R^{d}$ . Here $x_{n,t}^{m}$ is the vector of three coordinates of the *m* − th particle. The shape space variable describes population-level shape statistics, and is denoted as **Z**. As explained in later sections, this variable is used differently in the proposed and comparison methods. In general, a single random variable $Z\in R^{dM}$ is used to denote the vector form of the PDM for a subject at a specific time point, where coordinates from all particles are concatenated in a single vector.

### 2.2 Baseline methods

#### 2.2.1 Cross-sectional

Cross-sectional denotes the vanilla PDM optimization approach formulated for non-temporal modeling introduced in Cates et al. (2007), Cates et al. (2017). This is applied to spatiotemporal SSM by treating each time point as an independent observation, ignoring inter-subject correlation. We consider this baseline to showcase the impact of the sample independence assumption to model study designs with repeated measurements. Shape can be represented either as mesh or binary image volume, and the structure of the shape representation can vary across the cohort. Particle positions are constrained to shape surfaces and optimized by minimizing the following entropy-based objective.

$$Q_{\mathrm{cross}-\mathrm{sectional}}=\alpha H\left(Z\right)-\overset{N}{\underset{n=1}{\sum }}\overset{T}{\underset{t=1}{\sum }}H\left(X_{n,t}\right)$$ (1)

where *α* is a relative weighting parameter and *H* is the differential entropy. Minimizing this objective balances two terms. The first encourages a compact distribution of samples in the shape space, ensuring maximal correspondence between particles across shapes (i.e., lower model complexity). Minimizing this term alone would cause the particles to collapse to a single location on all shapes, providing the most compact model possible. The second encourages the maximal uniformly-distributed spread of points across individual shapes so that the shape is faithfully represented (i.e., better geometric accuracy). Intuitively the second term causes the particles on a given shape to repel each other and spread out. The combination of the two entropy terms encourages particles to spread uniformly across each shape while retaining geometrically consistent locations across shapes.

Eq. (1) is optimized *via* gradient descent. This requires taking the derivative of *H*(**Z**) with respect to particle positions. Differential entropy is defined as:

$$H\left(Z\right)=-\int_{Z}p\left(z\right)\log p\left(z\right)dz=-E\left[\log p\left(Z\right)\right].$$ (2)

To make this tractable, *p*(**Z**) is modeled parametrically as a Gaussian distribution with a population-specific mean ***μ*** and covariance matrix **Σ**. Assuming the shape space is Gaussian distributed introduces a generative statistical model:

$$z=\mu +\epsilon ,\epsilon ∼N\left(0,\Sigma \right)$$ (3)

where ***ϵ*** is a normally-distributed error. The entropy can then be estimated by:

$$H\left(Z\right)\approx \frac{1}{2}\log\Sigma =\frac{1}{2}\overset{dM}{\underset{i=1}{\sum }}\log\lambda_{i}$$ (4)

where *λ*_*i*_ are the eignevalues of **Σ**. The covariance is estimated from the data and found to be:

$$\frac{\partial H\left(Z\right)}{\partial X}\approx Y{\left(Y^{⊤}Y+\alpha I\right)}^{-1}$$ (5)

where **Y** denotes the matrix of points minus the sample mean ***μ*** of the ensemble, and the regularization term, *α*, accounts for the possibility of diminishing gradients (see Cates et al. (2007) for more detail). We get an update for each point by combining Eq. (5) with the shape-based updates explained in Cates et al. (2007). By intermittently fitting N(μ,Σ) to **Z** and updating the particle positions to decrease **Σ** (*via* Eq. (5)), *H*(**Z**) is minimized producing correspondence.

#### 2.2.2 Disentangled

Disentangled denotes the spatiotemporal SSM method proposed in Adams et al. (2022). This approach uses an adjusted optimization objective that disentangles the shape space entropy for **Z**_*t*_ and **Z**_*n*_, where **Z**_*t*_ represents shapes across subjects at a specific time point *t* (i.e., inter-subject variable) and **Z**_*n*_ represents shape across time for a specific subject *n* (i.e., intra-subject variable):

$$Q_{\mathrm{disentangled}}=\alpha \left(\overset{T}{\underset{t=1}{\sum }}H\left(Z_{t}\right)+\overset{N}{\underset{n=1}{\sum }}H\left(Z_{n}\right)\right)-\overset{N}{\underset{n=1}{\sum }}\overset{T}{\underset{t=1}{\sum }}H\left(X_{n,t}\right)$$ (6)

The first term encourages intra-subject correspondence across time points, the second promotes inter-subject correspondence across sequences, and the third retains geometric accuracy across subjects and time points. The particle updates are found in the same manner as in the cross-sectional formulation, except *p*(**Z**_*t*_) and *p*(**Z**_*n*_) are modeled as separate Gaussian distributions with covariance matrices **Σ**_*t*_ and **Σ**_*n*_, respectively. Thus the gradient has multiple terms that follow the form of Eq. (5). Given that the Gaussian distributions are fit using overlapping samples, simultaneously minimizing *H*(**Z**_*t*_) and *H*(**Z**_*n*_) encourages correspondence across all subjects and time points. In this approach, the inter- and intra-subject variability are disentangled. However, by assuming *p*(**Z**_*t*_) is Gaussian, the independence assumption is violated, and the temporal trajectory is diminished.

#### 2.2.3 Linear regression

Linear regression denotes the approach to spatiotemporal SSM presented in Datar et al. (2009) where regression analysis is incorporated into in the optimization process. This approach utilizes the same cross-sectional optimization objective (Eq. (1)) but optimizes correspondence with regression against an explanatory variable, *t*. This is done by replacing ***μ*** in the generative model of Eq. (3) with a function of *t*. The linear regression generative model can be written as:

$$z=f\left(t\right)+\tilde{\epsilon },\tilde{\epsilon }∼N\left(0,\tilde{\Sigma }\right)$$ (7)

where

$$f\left(t\right)=a+bt.$$ (8)

and $a\in R^{dM}$ are fit intercepts and $b\in R^{dM}$ are fit slopes. In the cross-sectional formulation, minimizing *H*(**Z**) decreases the entropy associated with ***ϵ***, which is the difference from the mean. Here, minimizing *H*(**Z**) decreases the entropy associated with $\tilde{\epsilon }$ , which is the residual from the linear regression model, *f*(*t*). Minimizing the residual has a similar effect to minimizing *H* (**Z**_*t*_) in the disentangled approach. Intuitively, we can think of *f*(*t*) as defining the mean of each *p* (**Z**_*t*_) distribution. By moving particles closer to *f*(*t*), we encourage inter-subject correspondence. However, unlike the disentangled approach, *f*(*t*) expresses linear time-dependency rather than diminishing intra-subject trajectory.

The linear parameters **a** and **b** are estimated by minimizing the sum of squared error (SSE) (Charnes et al. (1976)). Let *k* be an index for a shape of a specific subject at a specific time, then for shape $z_{k}\in R^{dM}$ , let $t_{k}\in R^{1}$ denote the time. The SSE is then defined as:

$$SSE=\underset{k}{\sum }{\left(z_{k}-f\left(t_{k}\right)\right)}^{2}$$ (9)

Estimating *via* a least-squares fit to the correspondence data,

$${\mathrm{argmin}}_{a,b}E\left(a,b\right)=\underset{k}{\sum }{\left[\left(a+bt_{k}\right)-z_{k}\right]}^{⊤}{\tilde{\Sigma }}^{-1}\left[\left(a+bt_{k}\right)-z_{k}\right].$$ (10)

The regression parameters are found to be:

$$a=\frac{1}{M}\left(\underset{k}{\sum }z_{k}-\underset{k}{\sum }bt_{k}\right)$$ (11)

$$b=\frac{\sum_{k}t_{k}z_{k}-\sum_{k}t_{k}\sum_{k}z_{k}}{\sum_{k}t_{k}^{2}-{\left(\sum_{k}t_{k}\right)}^{2}}$$ (12)

The regression optimization algorithm is then carried out as follows. Initial correspondences are optimized using the cross-sectional approach, and initial estimates for **a** and **b** are computed. Then correspondence positions are updated by replacing **Σ** from the cross-sectional formulation with $\tilde{\Sigma }$ , the covariance of the underlying residual relative to the generative model. The two estimation problems are then interleaved and the parameters **a** and **b** are re-estimated after each iteration of the gradient descent on the particle positions.

### 2.3 Proposed method: Regularized principal component polynomial regression

We propose to capture the trajectory of shape across time (from 1 to *T*) using polynomial regression. This could be accomplished similarly to the linear regression formulation by replacing the function *f*(*t*) in Eq. (7) with a polynomial. However, the spatial relationship between the particle coordinates is ignored in the linear regression approach. Each value of the *dM*-dimensional parameters **a** and **b** are fit separately without utilizing spatial correlations between points on a shape. This is fundamentally equivalent to fitting *dM* individual functions, one for each particle coordinate. There is no smoothness constraint that reflects the natural spatial regularity prior for anatomies, where the regression models for neighboring particles should be encouraged to vary smoothly over anatomical surfaces. This can lead to particle miscorrespondences across time and increases the risk of individual regression models overfitting the data noise.

To address this issue, we propose performing principal component analysis (PCA) (Abdi and Williams (2010)) on the set of shape space variables, then fitting a regularized polynomial in the principal subspace that represents the data span. PCA provides an orthogonal projection of the high dimensional particle sets, $z_{k}\in R^{dM}$ , onto a lower dimensional linear space, $R^{NT}$ , such that the variance of the projected data is maximized. By formulation, the dimensions of the principal subspace are independent and uncorrelated; thus, defining separate polynomial functions for each principal component is justified. The principal subspace is parameterized by mean vector (denoted $\mu \in R^{dM}$ ), a diagonal matrix of eigenvalues (denoted $\Delta \in R^{NT\times NT}$ ), and a matrix of eigenvectors (denoted $U\in R^{dM\times NT}$ ). Note we are modeling the full data span by considering *NT* − 1 eigenvectors, such that all shape variability is preserved. The projection of an instance **z**_*k*_ is defined as **q**_*k*_ = **U** ^*⊤*^(**z**_*k*_ − ***μ***). These projected representations, or PCA scores, can be mapped back to shape space as follows: **z**_*k*_ = **Uq**_*k*_ + ***μ***.

We propose to define the generative model as:

$$z_{k}=g\left(t_{k}\right)+\tilde{\epsilon_{k}},\tilde{\epsilon }∼N\left(0,\tilde{\Sigma }\right)$$ (13)

where

$$g\left(t_{k}\right)=Uh\left(t_{k}\right)+\mu$$ (14)

*g* (*t*_*k*_) maps a time value to a particle set, $g\left(t_{k}\right):R^{1}\to R^{dM}$ , and *h*(*t*_*k*_) is a polynomial of degree *P*. $h\left(t_{k}\right):R^{1}\to R^{NT}$ models the shape trajectory in the principal subspace over time and is defined as:

$$h\left(t_{k}\right)=\beta_{0}+\beta_{1}t_{k}+\beta_{2}{\left(t_{k}\right)}^{2}+\ldots \beta_{P}{\left(t_{k}\right)}^{P}=\beta_{0}+\overset{P}{\underset{p=1}{\sum }}\beta_{p}{\left(t_{k}\right)}^{p}$$ (15)

where $\beta_{0}\in R^{NT}$ is the intercept and $\beta_{p}\in R^{NT}$ (where *p* ∈ [1, *P*]) are the coefficients of the polynomial. This formulation requires selecting the degree of the polynomial, *P* ∈ [1, *T* − 1]. If *p* = 1 then *h*(*t*_*k*_) is linear, and if *P* = *T* − 1 or greater, then the curve will polynomially interpolate all of the points, meaning if *N* = 1, it would perfectly fit with a residual of zero. Selecting *P* = *T* − 1 would allow the polynomial to be maximally expressive or flexible, reducing residuals. However, there is a risk of over-fitting. Cross validation could be used to directly tune *P*, however this is computationally expensive. Thus to ensure model generalizability, we add regularization that biases ***β*** values to be small and sparse. We employ elastic net regularization (Zou and Hastie (2005)), which adds an *L*_1_ and *L*_2_ penalty on coefficients to the SSE cost function:

$$\underset{k}{\sum }{\left(q_{k}-h\left(t_{k}\right)\right)}^{2}+r_{1}\underset{p}{\sum }\|\beta_{p}{\|}_{1}+r_{2}\underset{p}{\sum }\|\beta_{p}{\|}_{2}^{2}$$ (16)

where *r*_1_ and *r*_2_ are parameters that control the weight of the regularization terms. The *L*_1_ penalty imposes a sparsity prior on the coefficients and the *L*_2_ penalty encourages the coefficients to have small magnitude. This allows us to set *P* = *T* − 1 and fit only relevant coefficients while keeping the rest close to zero, also known as variable selection. Regularization is necessary for robust, generalizable polynomial regression. It prevents the *h*(*t*_*k*_) from over-fitting to lesser components in the principal subspace that capture mostly noise so that false time dependency is not incorporated into the particle updates. We utilize 5-fold cross validation to select the optimal values for the *r*_1_ and *r*_2_ weights each time Eq. (16) is fit.

Optimization is carried out using a similar alternating process as in the linear regression approach. First initial correspondences are fit using the cross-sectional formulation. Next the polynomial coefficients {***β***_0_, ***β***_1_, … ***β***_*T*−1_} are fit using Eq. (16) on the PCA scores of the initial correspondence points. Then correspondence positions are updated by replacing **Σ** from the cross-sectional formulation with $\tilde{\Sigma }$ , the covariance of the underlying residual relative to *g*(*t*_*k*_). The two estimation problems are then interleaved. PCA is performed (updating **U** and ***μ***) and ***β***-values are re-estimated after each iteration of the gradient descent on the particle positions.

The particle density is a parameter that depends on the complexity of the shape cohort. Simple, smooth shapes can be described by fewer particles than more complex, variable shapes. ShapeWorks utilizes a particle splitting strategy in optimization. Particles are added in a multi-step fashion by splitting each particle to produce a new, nearby particle at each step until the desired number of particles is reached. This coarse-to-fine optimization scheme speeds up convergence to an acceptable local minimum Cates et al. (2017). It also allows for selecting the number of particles empirically, by adding particles until the representation is deemed to capture enough details for the given application.

### 2.4 Evaluation metrics

#### 2.4.1 Population variation analysis

In a PDM shape model, particle positions capture the modes of variation present in the population. Principal component analysis (PCA) is commonly utilized in SSM analysis to reduce the complexity of high dimensional shape models. PCA enables visualization and interpretation of the population-level shape variation while preserving the information captured by the PDM. We can identify the dominant modes of variation in the population as the PCA modes that account for a large portion of the overall variance. We can then visualize these modes by computing the mean of the correspondences and deforming the mean along the dominant modes to plus or minus one standard deviation. Meshes are created for such visualizations by first finding the warp transform between particles of shape with a known mesh and the particles of interest, then applying that transform to the mesh to create a new mesh that provides a denser representation of the particles of interest. In the case of spatiotemporal SSM, we would expect that the dominant modes of variation shift smoothly over time.

#### 2.4.2 Time dependency analysis

Analysis of spatiotemporal SSM also requires evaluating how well the PDM captures the underlying time dependency. If we know the true form of the underlying time dependency function *f*, then we can perform regression on the particles and analyze the *R* ^2^ value:

$$R^{2}=\frac{\sum_{k}\left(f\left(t_{k}\right)-\bar{z}\right)}{\sum_{k}\left(z_{k}-\bar{z}\right)}$$ (17)

The best possible *R* ^2^ score is one, indicating the regression equation explains all of the variability of the data. A constant model *f*(*t*) that always predicts the average of the particles, disregarding input time *t*, would get and *R*_2_ score of zero. If form of the underlying time dependency is unknown, we can utilize statistical tests to analyze the significance of the shape dynamics captured by the PDM.

## 3 Results

This section provides experiments that illustrate and validate the proposed method. First, we validate the method with synthetically generated *ellipsoids* (3D surfaces for which all plane cross sections are either ellipses or circles). Next, we present an application of real dynamic motion; the left atrium over the cardiac cycle. An overview of these datasets is provided in Table 1. ShapeWorks Cates et al. (2017) was used for cross-sectional optimization and as a starting point for implementing the proposed and other comparison methods.

**TABLE 1:** Overview of shape cohorts used in experiments.

Dataset	Number of subjects	Time points per subject	Number of particles	Optimization iterations	Covariance calculation frequency
Ellipsoid	30	8	128	1000	Every iteration
Left Atrium	28	25	1024	1000	Every 3rd iteration

### 3.1 Synthetic experiment

#### 3.1.1 Ellipsoid data generation

Synthetic shapes are useful in analyzing the performance of spatiotemporal PDM generation because the shape dynamics are formulated in a known way. We select to use a cohort of axis-aligned ellipsoids with differing *x*- and *y*-diameter values and a population consistent *z*-diameter value. The *x*-diameter represents a subject-dependent parameter that varies between subjects but not across time. The *y*-diameter represents a time-dependent parameter that varies across time in the same way for each subject. The *x*-diameter is randomly sampled for each subject from the following normal Gaussian distribution:

$$x-\text{diameter}∼N\left(0.6,0.1\bar{3}\right)$$ (18)

This results in *x*-diameters with a high probability (99.7%) of being in the range (0.2, 1). The *y*-diameter is defined to vary sinusoidal over time to mimic non-linear dynamics encountered in organ cycles. For each time point, *t*, the *y*-diameter is defined as follows for all ellipsoids in the cohort:

$$y-\text{diameter}\left(t\right)=0.6+0.4\sin\left(\frac{2\pi }{T}t\right)$$ (19)

**FIGURE 3:**
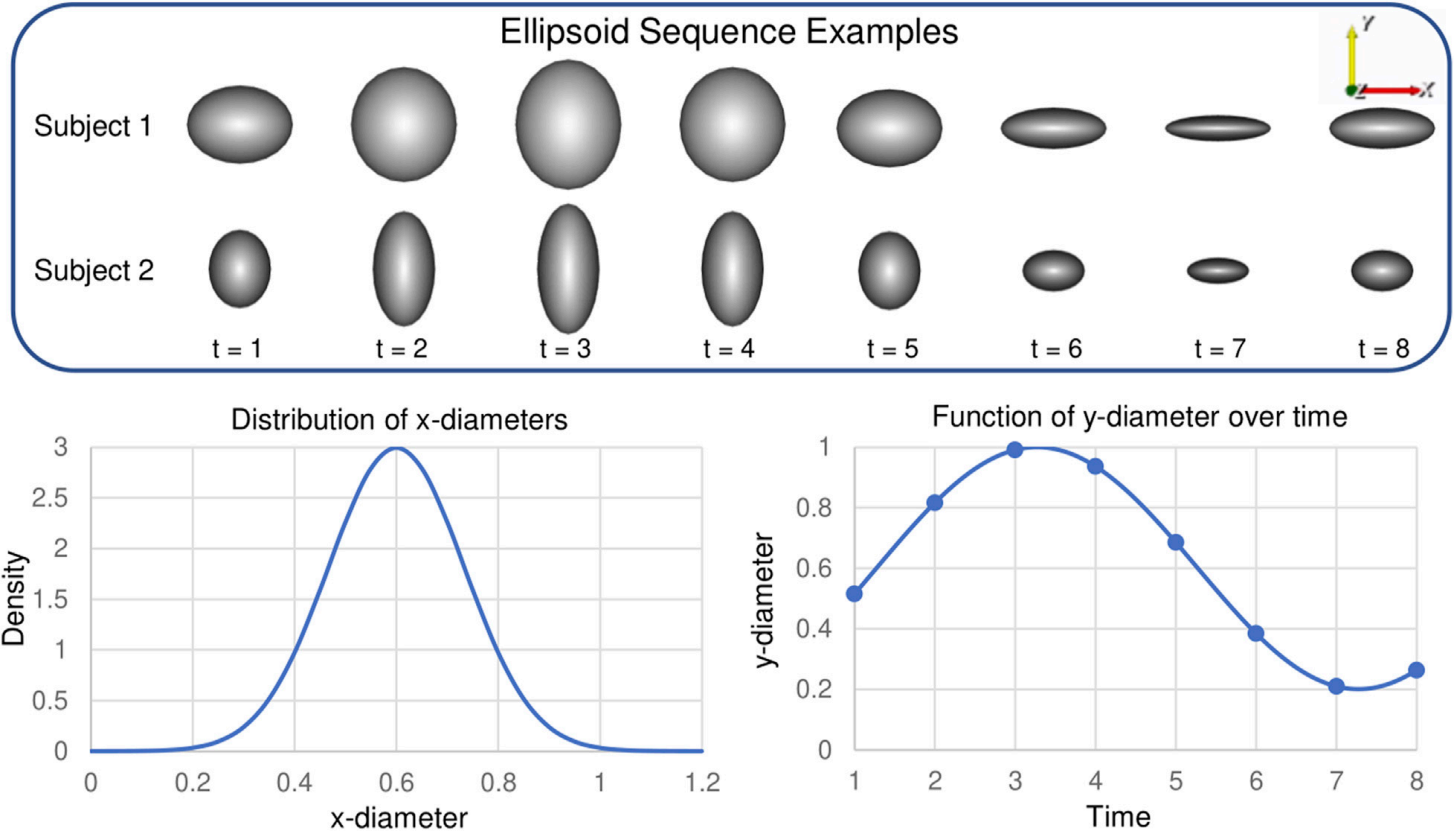
Synthetic Ellipsoid Data: Two example ellipsoids shape sequences are provided. Plots illustrate the distribution of subject-dependent *x*-diameters (Eq. 18) and the function for time-dependent *y*-diameters (Eq. 19).

This results in *y*-diameter values that vary cyclically between 0.2 and 1. We select the period and total time points to be *T* = 8. The *z*-diameter is fixed to be 1 across subjects and time for simplicity and 2D visualization purposes. These constraints result in ellipsoids with *x*, *y*, and *z*-diameters ≤1 ; thus all shapes fit within a unit cube. This scaling allows us to interpret SSE as relative SSE. Figure 3 displays plots of Eqs 18 and 19 as well as two examples of ellipsoid shape sequences.

#### 3.1.2 Ellipsoid results

We chose to use 128 particles in generating the PDM; this is sufficient for representing the simple ellipsoid shapes (see Figure 1).

##### 3.1.2.1 Modes of variation

As explained in Section 2.4.1, PCA is used to analyze and visualize the modes of variation captured by a PDM. Based on the construction of the ellipsoid cohort, a successful spatiotemporal shape model needs to meet the following requirements:

1. Overall variation should be described by two modes: the *x* and *y*-diameter.
2. For any given subject, inter-subject variation should be described by a single mode: the *x*-diameter.
3. At any given time point, intra-subject variation should be described by a single mode: the *y*-diameter.

**FIGURE 4:**
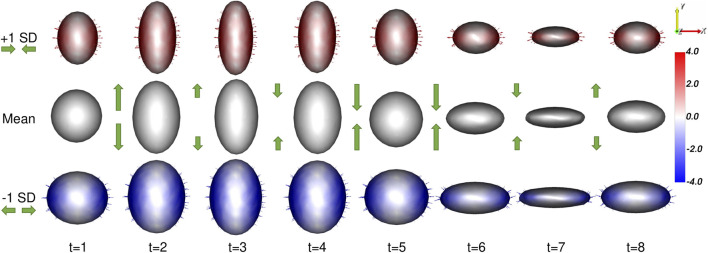
Ellipsoid Mode of Variation: The primary mode of variation from the proposed shape model is shown at each time point *via* the mean shape and ±1 standard deviations. The heat map and vectors show the difference to the associated mean. The subject-dependent *x*-diameter variation is correctly captured by the primary mode at each time point. The difference in the mean shapes across time correctly captures the time-dependent *y*-diameter. Green arrow annotations are provided to illustrate these dynamics.

The proposed method results in a PDM that meets all of these requirements. A visualization of the significant mode of variation at each time point is provided in Figure 4.

##### 3.1.2.2 Time dependency analysis

A spatiotemporal PDM should correctly capture the shape dynamics or underlying time dependency. In the case of the ellipsoid data, the dynamics are parameterized by a known sine function (Eq. (19)). Thus an ideal PDM could be expressed *via* a sine function for each subject. To test this, we use sinusoidal regression to fit subject-wise functions to the PDMs resulting from the proposed method and then quantify the residual error or SSE with respect to the fit functions. The general sine function:

$$s\left(t\right)=o+A\sin\left(2\pi ft\right)$$ (20)

is fit by estimating the parameters {*o* (offset), *A* (amplitude), and 2*πf* (period)} using least squares. The SSE resulting from subject-wise sinusoidal regression was 1.176e − 3 ± 2.468e − 3. The *R* ^2^, value (Eq. (17)) was found to be **0.999**, suggesting the proposed approach captured the time dependency very well.

### 3.2 Left atrium experiment

#### 3.2.1 4D left atrium data

**FIGURE 5:**
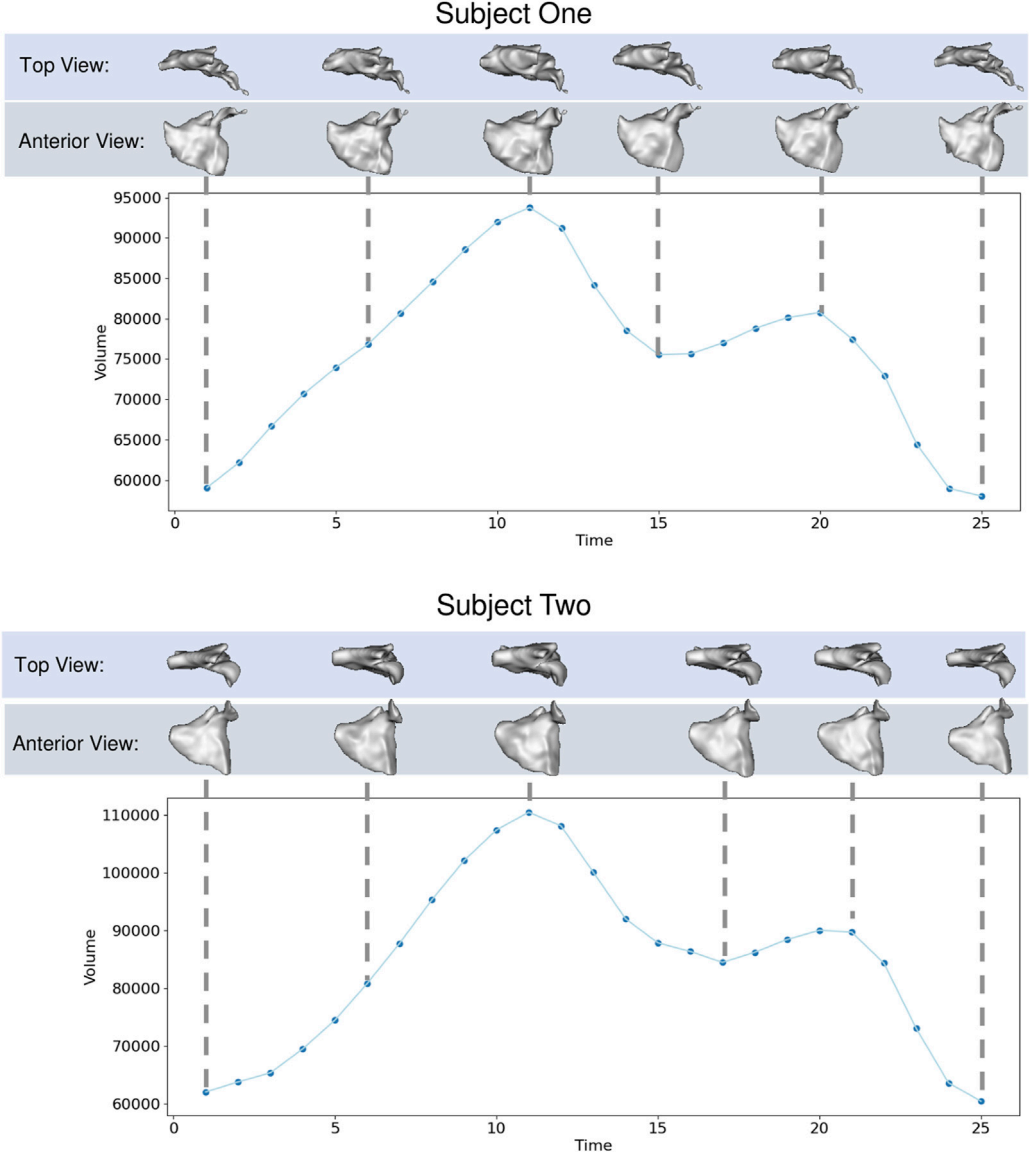
Left Atrium Examples: Left atrium volume is plotted over time for two subjects with meshes shown at selected time points from top and anterior view.

The left atrium shape cohort originated from 3D LGE and stacked CINE CMR scans of 28 patients presenting with atrial fibrillation between 2019 and 2020. The average patient age was 64.9 years, with 15 male and 13 female patients in the cohort. The scans for each patient used in this work were captured before a cardiac RF ablation procedure. Each CINE scan contained 25-time points covering the cardiac cycle (between R wave peaks). The temporal dimension was normalized at the time of acquisition to cover one heartbeat for each patient. Thus the number of milliseconds covered varies patient-wise. The 3D LGE images were manually segmented by a cardiac imaging expert, and this segmentation was matched to the closest CINE time-point based on CMR trigger time. The segmentation was then transformed to each time point through pairwise deformable registrations to create a full 3D segmentation for each time point Morris et al. (2020). This technique reduces the manual burden and has been shown to produce segmentations with a reasonable error when compared to fully manual segmentation Parikh et al. (2019). An example of one subject left atrium mesh sequence is provided in Figure 5.

**FIGURE 6:**
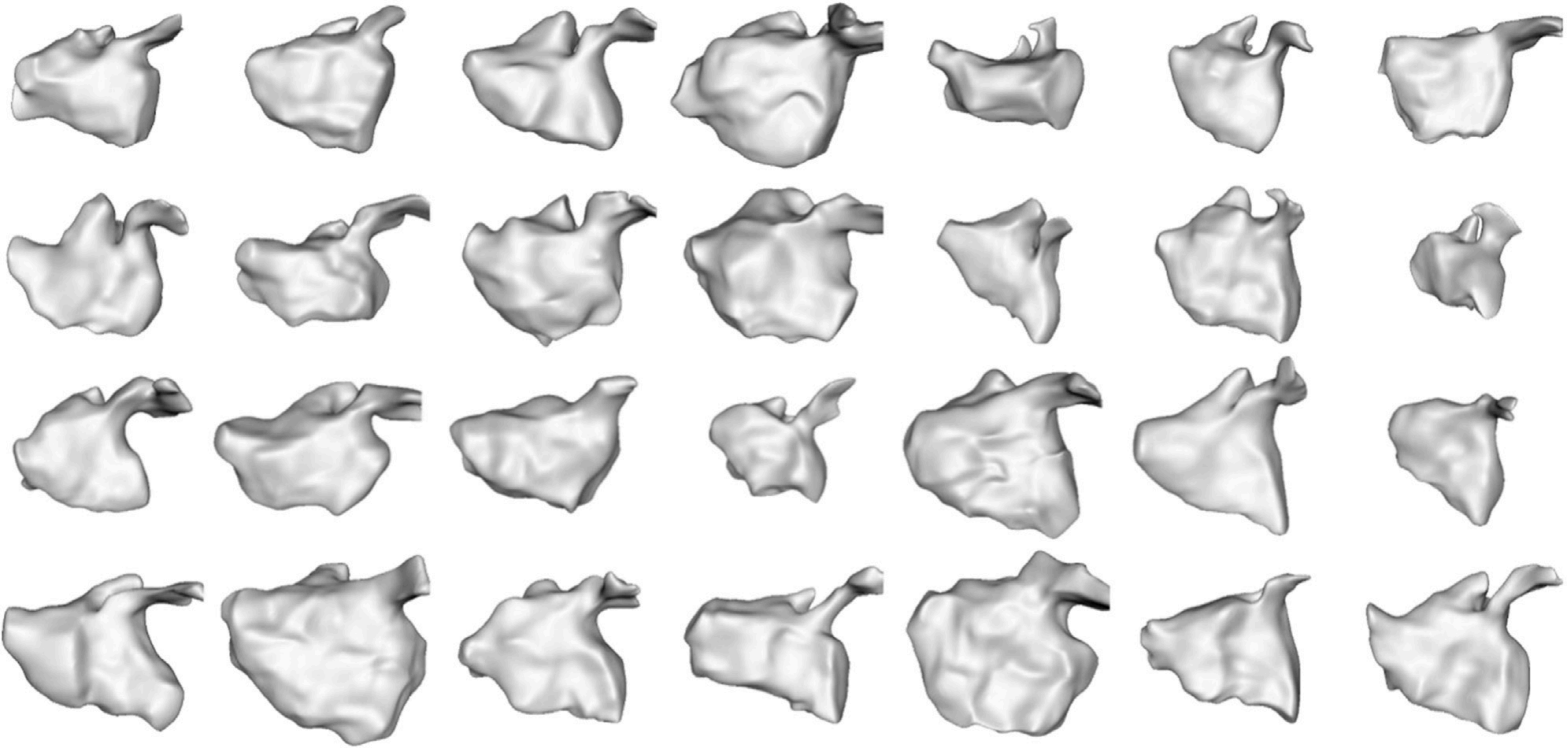
Left Atrium Subjects: The first time point mesh for all 28 subjects in the left atrium cohort is displayed from the anterior view. The left atrium shape is highly variable across subjects.

We selected this dataset to demonstrate how the proposed approach can correctly capture highly non-linear dynamic motion. The left atrium shape varies greatly across patients (see Figure 6), and atrial fibrillation affects the non-linear dynamics in differing ways. These challenges help demonstrate the robustness of the proposed spatiotemporal modeling approach.

#### 3.2.2 Left atrium results

**FIGURE 7:**
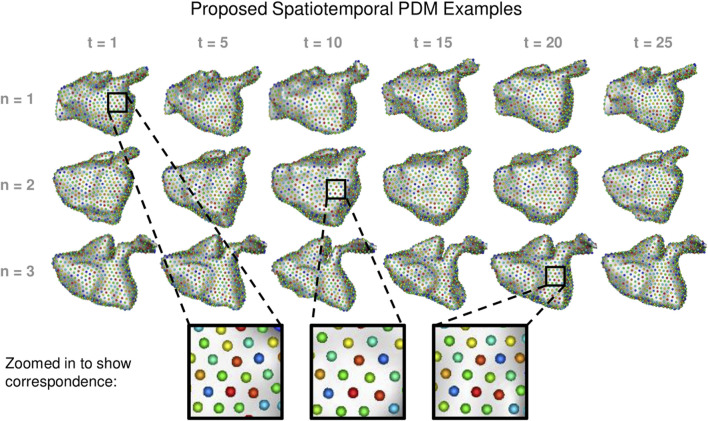
Proposed Spatiotemporal PDM Examples: Particles from the proposed approach are shown at selected time points for three different subjects. Zoomed-in boxes illustrate that particle correspondence (denoted by color) is maintained across subjects and time.

We built a PDM using the proposed method as well as the cross-sectional, disentangled, and linear regression comparison methods. We used 1024 particles in each left atrium PDM, which is sufficient to capture the details of the shapes. Examples of the PDM resulting from the proposed method are provided in Figure 7. Particle correspondence was maintained across both time points and subjects.

**FIGURE 8:**
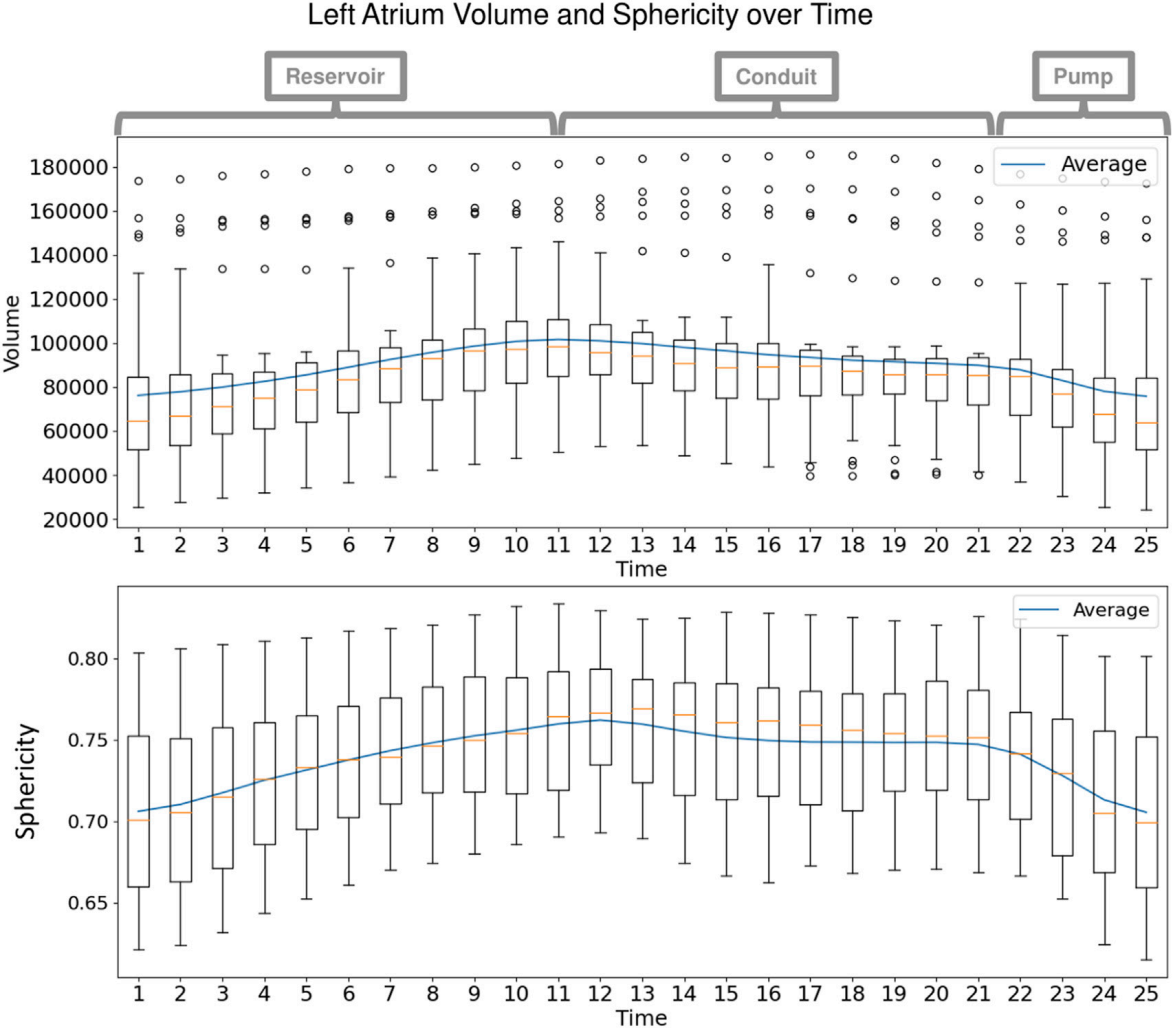
Left Atrium Volume over Time: Box plots display the spread in volume of the ground truth left atrium meshes across the subjects at each time point in the cardiac cycle. The mean volume is plotted as a blue line across time. Annotations at the top show the intervals of the three left atrium functions: reservoir during ventricular systole, conduit during early diastole, and pump during end diastole.

**FIGURE 9:**
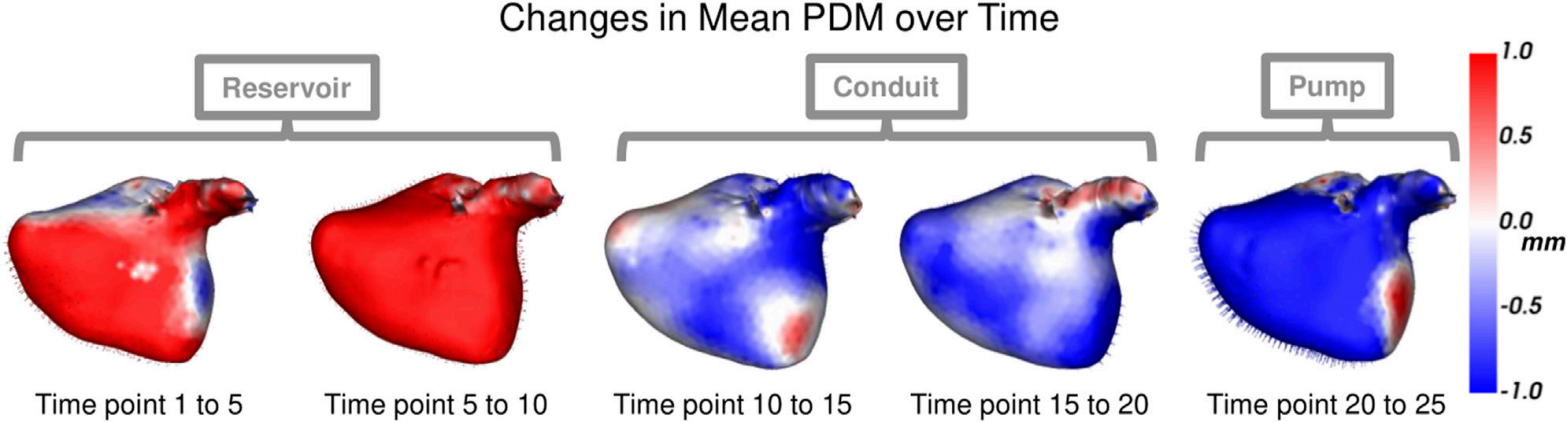
Mean Shape Dynamics: The mean shape from the proposed PDM is shown from the anterior view at a subset of time points. Heat maps show the change in shape to the next displayed time point mean shape. Here red denotes expansion, and blue denotes contraction. The mean shape dynamics correctly match the mean volume over time (Figure 8) and three left atrium function intervals: reservoir, conduit, and pump.

Each left atrium shape sequence covers one heartbeat, from the peak of 1 R wave to the peak of the next R wave. The spread of the volumes of the left atrium meshes across time is visualized *via* box plots in Figure 8. Here we can see the three key left atrium functions: reservoir or filling (where the volume increases), conduit or passive emptying (where volume decreases slowly in a decelerating manner), and pump or active emptying (where volume decreases quickly). Figure 9 shows the mean shapes resulting from the proposed PDM at five-time points. Heat maps show the difference to the next time point mean, where red indicates expansion and blue indicates contraction. Here we can see that the mean shapes correctly expand during the reservoir, slightly contract during the conduit, and further contract during the pump. This demonstrates that the proposed method correctly captures the non-linear dynamics of the left atrium across the cardiac cycle.

##### 3.2.2.1 Modes of variation

**FIGURE 10:**
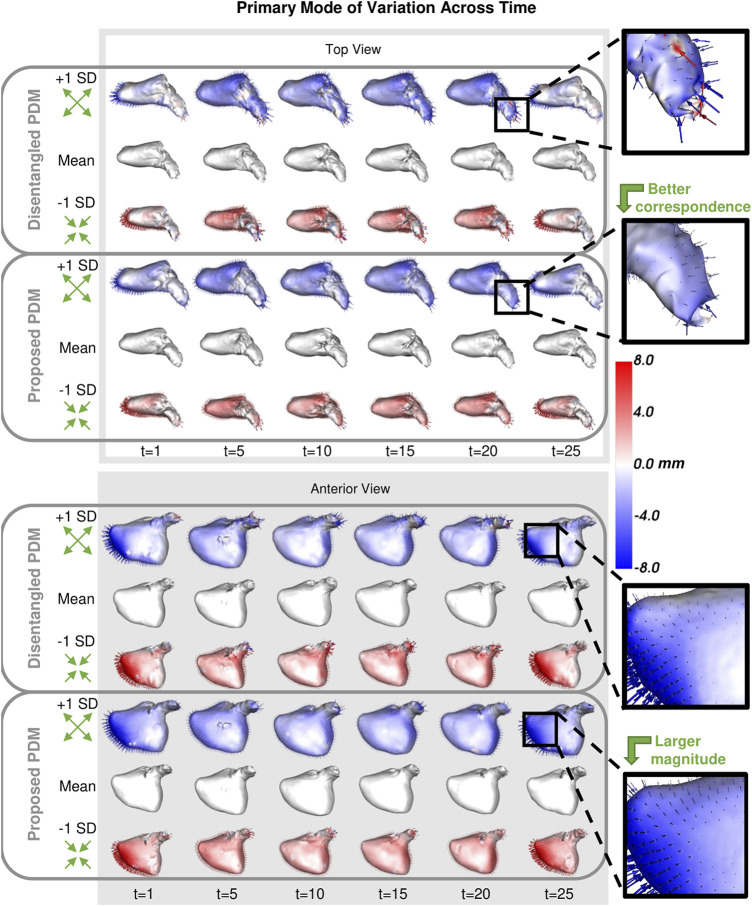
Primary Mode of Variation over Time: The primary mode of variation shown at a subset of time points for the disentangled PDM and proposed PDM shown from the top and anterior view. The heat map and vectors show the difference to the associated mean. Green arrow annotations are provided to note the change, and zoomed-in boxes on the left help illustrate some of the differences.

As with the synthetic data experiment, we utilize PCA to analyze and visualize the modes of variation captured by the PDMs. We are interested in whether the PDM correctly captures the primary mode of variation at each time point. This primary mode is expected to be overall size or *sphericity* given the large spread of left atrium volume across subjects (Figure 8). Sphericity is calculated as $\frac{\pi^{1/3}{\left(6*\text{Volume}\right)}^{2/3}}{\text{Surface Area}}$ where a higher value indicates the shape is closer to a sphere (for a perfect sphere, sphericity = 1). In Figure 10, we display the primary mode captured by the disentangled and proposed approaches across time from top and anterior view.

##### 3.2.2.2 Time dependency analysis

For this experiment, we do not know the parametric form of the underlying time dependency as we did for the synthetic ellipsoid. Thus in order to analyze if the shape models are capturing the underlying time dependency, we must measure the statistical significance of the relationship between particle positions and time. Here we employ a *repeated measures ANOVA* test, which is used to determine whether or not there is a statistically significant difference between the means of multiple groups in which the same subjects show up in each group (Girden (1992)). In this case, the null hypothesis is that there is no difference in the mean particle positions at each time point. Rejecting this hypothesis means that at least one time-point mean is different from the rest, thus the PDM is capturing some time dependency. To conduct this test, we utilized the repeated measures function *RM()* from the R package MANOVA. RM (Friedrich et al. (2018)) with significance level 0.05, specifying both time and particle coordinate index as within-subject factors. For computational memory purposes, a consistent randomly selected subset of 100 particle coordinates is used. The *RM()* function calculates the modified ANOVA-type statistic (Friedrich and Pauly (2018)) for repeated measure designs with metric data. The assumptions of the multivariate repeated measures ANOVA test are met as follows:

• Random Samples: The subject that comprises the left atrium cohort are assumed to be a random sample from the population of interest.
• Independent Observations: The subject sequences in the left atrium cohort are independent of each other. Note that while shape sequences are assumed to be independent, shapes within a given sequence are not, they are considered repeated measures.
• Multivariate Normality: There are normally distributed population values for each particle position at each time point. This was verified using the Shapiro-Wilk test, which provided p-values greater than 0.05 in all cases.
• Sphericity: As we are performing a repeated measures ANOVA factor with two levels (time and particle coordinate), the sphericity assumption is automatically met.

In Table 2, we report the test statistics and corresponding *p*-values for the PDM resulting from each of the optimization approaches.

**TABLE 2:** Repeated measures ANOVA-type test statistic (larger is better, in bold) and corresponding *p*-value (smaller is better, in bold).

	Test statistic	*p*-value
Cross-Sectional	2.272	0.114
Disentangled	4.368	0.019
Linear Regression	2.568	0.090
Proposed	**9.186**	< **0.001**

### 3.3 Discussion

The synthetic ellipsoid experiment provides a proof-of-concept, demonstrating the efficacy of our proposed method. The resulting PDM correctly captured the inter-subject variation as the *x*-diameter (Figure 4) and the intra-subject variation as the *y*-diameter. Additionally, the resulting PDM captured the known time dependency very well (with *R* ^2^ = 0.999). The left atrium dataset served as a real use case of dynamic organ motion. Each left atrium sequence was comprised of 25 time points which covered the span of one cardiac cycle, including the reservoir, conduit, and pump phases The time dependency underlying left atrium dynamics is not parameterized by a known function. However, it is known that throughout the cardiac cycle, the amount of blood contained by the left atrium changes, resulting in a change in volume and sphericity (Figure 8). Figure 9 shows that the changes in the mean shapes resulting from the proposed approach correctly capture this underlying mechanism. In mode visualization (Figure 10), we selected not to compare against the cross-sectional and linear regression approaches as these approaches are ill-suited to capture the non-linear dynamics (as is evident by the subsequent time dependency analysis). Both the PDM generated by disentangled optimization and the proposed optimization correctly capture the primary mode as size. However, the primary mode from the proposed approach is more consistent across time and demonstrates better correspondence. This is evident by the smooth deformation from the mean to ±1 standard deviation at each time point. The primary mode from the proposed approach also explains a larger proportion of the overall population variability than the cross-sectional, indicating a superior, more compact model.

The statistical test demonstrated that the proposed approach captured the underlying time dependency better than the baseline methods (Table 2). In the case of the cross-sectional and linear regression models, the *p*-values are greater than 0.05; thus, we accept the null hypothesis that there is no difference in the mean particle positions at each time point. The cross-sectional and linear regression approaches do not provide a PDM that captures the time dependency. The disentangled and proposed models, in contrast, provide enough evidence to reject the null hypothesis, suggesting that they are capturing the time dependency. Furthermore, the proposed PDM resulted in a larger test statistic and a *p*-value that is an order of magnitude smaller than the disentangled PDM. This suggests the time dependency is more strongly captured by the proposed model. These experiments validate the efficacy of the proposed approach in modeling non-linear dynamic shape and surpassing the limitations of existing spatiotemporal SSM methods.

#### 3.3.1 Limitations

The proposed approach and baseline comparison methods inherit the limitations of particle-based shape modeling. One such limitation is defining correspondence with respect to topological changes within a shape population. The proposed approach assumes that shapes within the cohort have similar features across time and subjects. Addressing anomalies or sub-groups with the cohort would require additional methodology that is out of the scope of this work. An additional limitation is this approach is not generative. While partial shape sequences can be use in PDM optimization, this model is not capable of inferring the missing time points of a subjects sequence.

#### 3.3.2 Future work

In future work, this formulation could be extended to utilize regularized non-linear mixed effect modeling in the principal subspace rather than regression. This hierarchical approach would provide the benefit of characterizing both individual subject trends and an overall population trend. Alternatively, we could utilize a time-series generative statistical model for modeling the shape projections, such as the linear dynamical system. This generative approach would allow for inferring shapes for missing time points in subject sequences.

## 4 Conclusion

We presented a principled approach for statistical shape modeling of non-linear dynamic anatomies. By incorporating regularized principal component polynomial regression into the PDM optimization scheme, we are able to capture the underlying non-linear shape trajectories in a smooth, generalizable manner. We demonstrated our approach on synthetic ellipsoids as a proof-of-concept and verified that it outperforms existing methods of spatiotemporal SSM on a real cohort of left atrium over the cardiac cycle. Our approach results in SSM with inter and intra-subject correspondence that correctly captures a statistically significant underlying time dependency. Additionally, our approach does not require temporal sequences to be consistent across subjects, allowing for the use of partial observations or missing time points. Alleviating the requirement of complete sequences makes the approach more viable as medical shape data is typically scarce. Spatiotemporal SSM has great potential to inform clinical research regarding dynamic anatomy and longitudinal shape changes. Our approach provides a principled solution for capturing non-linear shape trajectories, greatly increasing the potential for SSM utilization in clinical studies.

## Data Availability

The data analyzed in this study is subject to the following licenses/restrictions: The left atrium dataset was provided by the University of Utah Division of Cardiovascular Medicine and is not publically available. Requests to access these datasets should be directed to AM, alan.morris@utah.edu.
